# Immune Checkpoint Inhibitors and Their Cardiovascular Adverse Effects

**DOI:** 10.3389/or.2023.11456

**Published:** 2023-11-17

**Authors:** Ravi Kumar Paluri, Yochitha Pulipati, Dileep Kumar Reddy Regalla

**Affiliations:** ^1^ Department of Hematology-Oncology, Atrium Health Wake Forest Baptist, Winston-Salem, NC, United States; ^2^ Department of Internal Medicine, Allegheny General Hospital, Pittsburgh, PA, United States; ^3^ Department of Hospital Medicine, OSF Saint Anthony Medical Center, Rockford, IL, United States

**Keywords:** immune checkpoint inhibitor, myocarditis, pericarditis, ICI rechallenge, glucocorticoids

## Abstract

Immune checkpoint inhibitors (ICIs) have reshaped and have become a well-established treatment modality for multiple advanced-stage malignancies. ICIs block the immune system regulatory checkpoints, namely CTLA-4 and PD-1/PDL1, which provokes excess immune response against self-antigens. Immune modulation with ICIs can result in diverse immune-related adverse events targeting organ systems. Several cases of ICI-related cardiotoxicity were reported, while the actual incidence was likely underestimated due to heterogeneous clinical presentation. These include, but are not limited to, myocarditis, pericarditis, atherosclerosis, and arrhythmia. EKG, Troponin, Echocardiogram (TTE), and Cardiac MRI (CMRI) are indispensable diagnostic tools to aid in the management of cardiac adverse effects. Herein, we review the ICI-mediated cardiovascular adverse events, diagnosis, treatment strategies, and reintroduction of ICIs post-cardiotoxicity.

## Introduction

Immune checkpoint inhibitors (ICIs) have changed the landscape of management of several cancers harnessing anti-tumor adaptive immunity by inhibiting key immune system inactivators such as CTLA-4, PD-1, and PDL-1. However, the robust immune response could act against self-antigens leading to significant toxicity. Cardiovascular toxicity is one of the primary forms of toxicity that often leads to treatment discontinuation. Most of the evidence on cardiovascular toxicity of ICIs has been limited to case series and systematic reviews, and little was known about ICI re-challenge post-cardiotoxicity. This review will highlight the available evidence on the most common ICI-related cardiotoxicity and their reintroduction.

### Types of Cardiotoxicity

Ipilimumab (CTLA-4 inhibitor), Pembrolizumab/Nivolumab/Durvalumab (PD-1 inhibitors), and Atezolizumab (PD-L1 inhibitor) are the commonly used immune checkpoint inhibitors [1]. More recent ICIs include PD-1 inhibitors such as Dostarlimab [2] and Cemiplimab [3]. ICI-related cardiotoxicity has varied clinical manifestations, with myocarditis, pericarditis, cardiomyopathy, and arrhythmia being the commonly described ones. Other toxicities include vasculitis, hypertension, and atherosclerosis. A study has shown a three-fold higher risk of aortic plaque progression and coronary atherosclerosis leading to myocardial infarction and coronary revascularization [4]. Although myocarditis is potentially fatal, the treatment limitations of other cardiac manifestations have implications for further treatment continuation. According to a study, the most common cardiac complication was heart failure (17%), followed by myocarditis (15%) and pericarditis (13%) with myocarditis carrying the highest mortality rate [5]. Coronary artery vasculitis was reported in a few studies.

## Myocarditis

CTLA-4 and PD-1, which are co-inhibitory molecules on T cells [6], are presumed to have a homeostatic role in the myocardium based on the pre-clinical data. For example, loss of CTLA-4 or PD-1 in mice induces spontaneous myocarditis [7, 8]. CTLA-4 is innately present on regulatory T cells, and inhibiting it promotes T cell stimulation [9]. Evolving data suggests that blocking PD-1 from binding with PD-L1 on cardiac myocytes triggers T cells against the myocardium [9]. Johnson et al hypothesized that a selective T-cell clone might attack the myocardium due to a common antigen between the tumor and myocardium, similar to the shared antigen theory [10]. Another theory is the development of autoantibodies causing myocarditis while on ICIs, as evidenced by pathology showing autoantibodies in a study [11, 12]. An autopsy study demonstrated predominant CD4^+^ T cell infiltration in the heart of patients treated with CTLA-4 inhibitor compared to CD8^+^ T cell infiltration in those treated with a PD-1 inhibitor [13]. It was proposed that pre-exposure to chemotherapy or radiotherapy could liberate cardiac antigens leading to enhanced ICI-related cardiotoxicity [14].

Incidence and prevalence of myocarditis varied greatly between studies owing to misclassification bias and lack of timely cardiac monitoring. The true incidence is difficult to estimate. For instance, a study has shown that concomitant treatment with Nivolumab and Ipilimumab causes myocarditis in 0.27% of patients vs. 0.06% in patients receiving only Ipilimumab [10]. In contrast, another study estimated the prevalence to be 1.14% [15]. A study estimated that there has been a 42% increase in the patient pool who qualify for ICIs from 2011 to 2018 [16]. It is unclear if this widened patient pool leads to an increased prevalence of myocarditis. The average onset time was 17 days between receiving ICIs and the development of myocarditis [10], in contrast to another study, which found that the average onset time was 34 days ranging from 1–3 months [15]. Another retrospective study demonstrated an average emergence time of 27 days, with 76% of cases reported within 6 weeks of starting treatment [17]. It is unclear if exposure to a different ICI, before the actual ICI that triggered the myocarditis, decreases the onset time of myocarditis, as evident in a few case reports [18].

Also, concomitant administration of CTLA-4 and PD-1/PDL-1 inhibition and diabetes mellitus were distinct predisposing factors for the development of ICI-associated myocarditis [15]. It is unclear if the type of cancer, pre-existing cardiac pathology, and autoimmune diseases increased the risk of myocarditis [19–22]. In a systematic review involving 88 cases, dyspnea (49%), followed by fatigue (25%), and chest pain (17%) were the most commonly reported symptoms [23–26]. Interestingly, smoldering myocarditis with little to no symptoms was reported [27].

### Diagnosis of ICI-Related Myocarditis

In most cases of myocarditis, clinical picture and cardiac MRI (CMRI) help presume the diagnosis, though a gold standard procedure, endomyocardial biopsy (EMB) is seldom done due to its invasive nature [28].

Numerous definitions were proposed for the diagnosis of ICI-related myocarditis. Common terminology criteria for adverse events (CTCAE) has been historically used until Bonaca et al [29], put forward a criteria for standard assessments. More recently, the International Cardi-Oncology Society (IC-OS) proposed a criteria as in Table 1 below [30]. IC-OS criteria are binary, do not include PET scan, and rather include immune-related adverse events (irAEs) affecting other organ systems compared to criteria by Bonaca et al. In addition, a study has proposed including an onset time of symptoms of less than 3 months in the minor criteria to improve the specificity of IC-OS definition [31].

**TABLE 1:** [30] International Cardio-Oncology Society Consensus 2021 definition for immune checkpoint inhibitor myocarditis. Reprinted from defining cardiovascular toxicities of cancer therapies: An International Cardio-Oncology Society (IC-OS) consensus statement by Herrmann et al. (2021).

**Diagnosis**
Either pathohistological diagnosis: Multifocal inflammatory cell infiltrates with overt cardiomyocyte loss by light microscopy of cardiac tissue samples
Or clinical diagnosis^a^ ^,^ ^b^
A troponin elevation^c^ (new, or significant change from baseline) with 1 major criterion or a troponin elevation (new, or significant change from baseline) with 2 minor criteria after exclusion of acute coronary syndrome or acute infectious myocarditis based on clinical suspicion
**Major criterion**
CMR diagnostic for acute myocarditis (modified Lake Louise criteria)
**Minor criteria**
Clinical syndrome (including any one of the following: fatigue, muscle weakness, myalgias, chest pain, diplopia, ptosis, shortness of breath, orthopnea
Lower extremity edema, palpitations, light-headedness/dizziness, syncope, cardiogenic shock)
Ventricular arrhythmia and/or new conduction system disease
Decline in cardiac (systolic) function, with or without regional wall motion abnormality in a non-Takotsubo pattern
Other immune-related adverse events, particularly myositis, myopathy, myasthenia gravis
Suggestive CMR (meeting some, but not all, of the modified Lake Louise criteria)

^a^ Clinical diagnoses should be confirmed with cardiac magnetic resonance imaging (CMR) or endomyocardial biopsy if possible and without causing delays of treatment.

^b^ In a patient that is clinically unwell, treatment with immunosuppression should be promptly initiated while awaiting further confirmatory testing.

^c^ Both troponin I and troponin T can be used; however, troponin T may be falsely elevated in those with concomitant myositis.

EKG: No specific finding on EKG was diagnostic of ICI myocarditis and majority of case reports showed widely variable findings like T wave inversions, sinus tachycardia, non-specific ST-T changes, QT prolongation, and even Torsades [32]. An abnormal ECG was found in 89% of myocarditis patients in a study [15]. Also, the worst prognostic features such as high grade heart block and ventricular arrhythmias were found in 28% and 22% of patients [26].

### Cardiac Biomarkers

Troponin is crucial in the diagnosis of ICI-related myocarditis. Obtaining baseline troponin I is recommended in all patients before initiating ICIs [1]. A systematic review has shown elevated troponin in 42 out of 43 cases [26]. Normalization of troponin levels was observed in patients who responded to immunosuppressive therapy [27, 33, 34]. In contrast, an increasing troponin level is not always suggestive of or diagnostic of myocarditis, mandating comprehensive evaluation [35]. Nevertheless, ≥1.5 ng/mL troponin is linked to major adverse cardiac events [15]. As outlined in the IC-OS definition, troponin is a requisite for clinical diagnosis of ICI-related myocarditis. While studies suggested serial troponin monitoring for early diagnosis of ICI-related myocarditis, caution is advised in clinical interpretation due to significant false positives and inadvertent interruption in therapy [36]. In addition to troponin, CK-MB and CK levels were raised in many cases [18, 25, 26, 37]. Higher CK and CK-MB levels were linked to worse mortality [36]. Elevated BNP/NT pro-BNP [15, 25, 26, 43] and anti-striated muscle antibodies [19] are associated with the onset of myocarditis. Therefore, obtaining a baseline BNP/NT pro-BNP is recommended per European Society of Cardiology (ESC) guidelines [1].

#### Transthoracic Echocardiogram (TTE)

A multicentre registry showed preserved ejection fraction (EF) in 51% of cases [15], and it was unclear if preserved EF correlates well with a worse prognosis. A systematic review reported that 8 out of 13 cases with preserved EF died eventually [26]. It was also unclear if low EF leads to high mortality. In patients with a low risk of myocarditis, the global longitudinal strain could have a role, especially when EF is a poor diagnostic and prognostic indicator [38]. Also, a retrospective study of 101 patients showed that global longitudinal strain was lower in myocarditis compared to controls [39]. More recently, a study has shown that a reduction in longitudinal strain was linked to elevated troponin I levels [40]. A baseline TTE is recommended in patients who are at high risk of developing ICI-related myocarditis [1].

#### Cardiac MRI (CMRI)

CMRI is widely used to diagnose myocarditis. The characteristic findings include late gadolinium enhancement (LGE) with myocardial wall edema on T2-weighted imaging. A recent original research article involving six patients showed LGE in five out of six cases [18]. In contrast, another recent international registry study showed LGE in 48% of patients with ICI-associated myocarditis which warrants caution when relying on CMRI alone [41]. This low sensitivity could be explained by cases involving scattered to low-grade [40] myocardial inflammation [42].

#### Fluorodeoxyglucose-Positron Emission Tomography (FDG-PET)

Due to the lack of sensitivity of CMRI in cases with little to no myocardial inflammation, researchers have evaluated FDG-PET for its applicability. In a prospective study, FDG-PET complemented CMRI findings in 65 patients who were evaluated for possible myocarditis [43]. FDG-PET has been included in the diagnostic criteria by Bonaca et al.

### Endomyocardial Biopsy

Endomyocardial biopsy has been considered the gold standard technique in diagnosing myocarditis from ICIs; however, this may not be feasible in all clinical scenarios. In a systematic review of 26 cases (14 cases underwent biopsy during angiography and 12 cases during autopsy), the biopsy showed predominantly lymphocytic myocarditis along with other cells. CD8+ve T cells were the major group of lymphocytes, along with occasional CD4+ve T cells [26]. Autopsy in a few case reports showed CD3^+^ T cells [44]. An autopsy showed lymphocytic myocarditis with patchy fibrosis with a confirmation of non-infectious etiology [45]. Despite being a gold standard diagnostic study, EMB could miss the diagnosis in patients presenting with focal myocardial inflammation.

### Grades of Myocarditis

G1: Abnormal cardiac biomarker testing, including abnormal ECG.

G2: Abnormal screening tests with mild symptoms.

G3: Moderately abnormal testing or symptoms with mild activity.

G4: Moderate to severe decompensation, IV medication or intervention required, life-threatening conditions [46].

### Management

The treatment of ICI-associated myocarditis is extrapolated from non-immunotherapy-related myocarditis. For example, ICI-associated myocarditis commonly presents in older populations, more likely to be associated with ventricular tachycardia/ventricular fibrillation/advanced heart block and higher mortality when compared to non-ICI myocarditis. Nevertheless, advanced age, co-morbidities, and advanced cancer could explain higher mortality [47].

**FIGURE 1:**
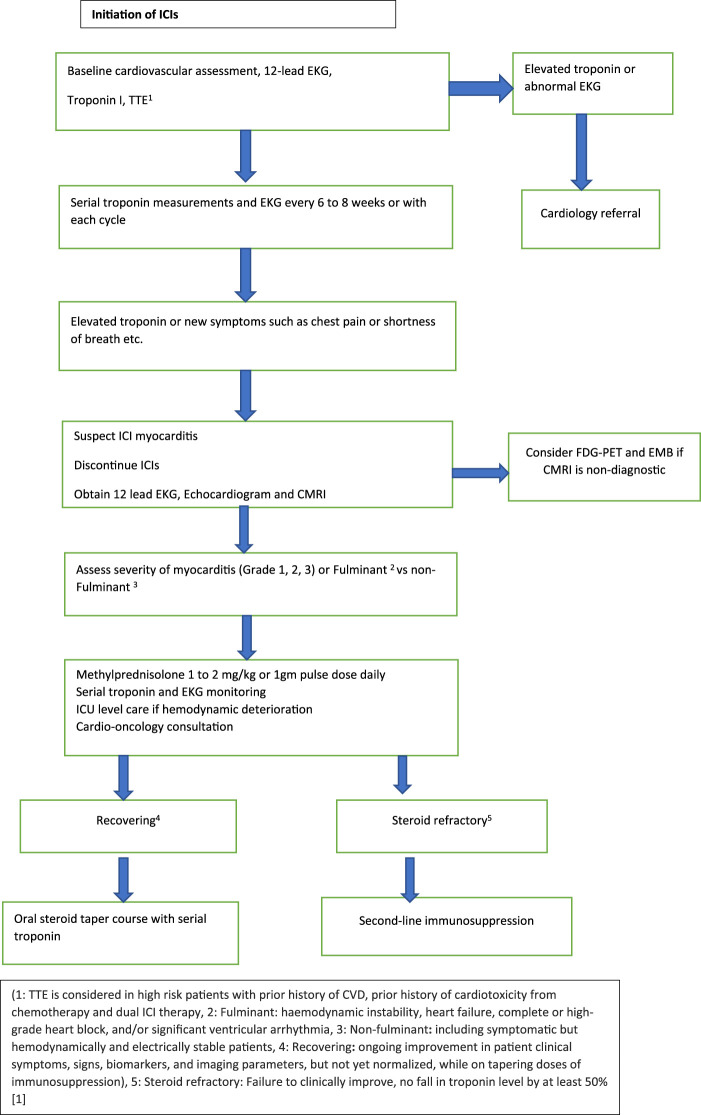
Management of ICI associated myocarditis.

Most case reports and systematic reviews initiated Methylprednisolone 1–2 mg/kg when myocarditis was suspected. In patients who worsen, few studies suggested the addition of Mycophenolate mofetil (MMF) or tacrolimus [48]. Also, in patients with limited response to initial doses of steroids, Methylprednisolone 1 gm/day can be considered [46]. Some experts and a few case reports have shown the benefit of using methylprednisolone 1 g daily upfront especially in patients in which high troponin corresponds to a higher risk of significant cardiac adverse effects [18, 19]. NCCN guidelines recommend a methylprednisolone pulse dose of 1 gm/day for G3 and G4 myocarditis, and steroid tapering over 4–6 weeks until the cardiac function returns to baseline [49]. ASCO guidelines recommend holding ICI for G1 and rechecking troponins 6 h after initial elevation. Resuming ICI could be considered after normalization of troponin. For Grade ≥2 myocarditis, ASCO recommends holding and initiating high-dose corticosteroids (1–2 mg/kg/day) within 24 h. In patients without an immediate response, pulse dose methylprednisolone 1g every day should be initiated [50]. Gradual tapering over 4–6 weeks is instituted when cardiac function returns to baseline. The flow chart (Figure 1) presented is derived from preceding data and expert consensus [50, 51].

### Second-Line Immunosuppression

Various immunosuppressants have been studied for steroid-refractory cases. Steroid refractory myocarditis is defined as rising troponin or <50% reduction in troponin from peak with no clinical improvement [51]. NCCN recommends adding Antithymocyte globulin (ATG) or infliximab for G4 myocarditis if no improvement is seen within 24 h on steroids. However, it is important to note that infliximab is contraindicated in heart failure [49]. Limited evidence also suggests elevated cardiovascular death risk with infliximab [52]. In contrast, ESMO guidelines recommend adding tocilizumab or mycophenolate mofetil (MMF) as second-line agents followed by ATG or alemtuzumab or abatacept as third-line agents [51]. Additionally, ASCO guidelines recommend abatacept or alemtuzumab as additional options for life-threatening cases [50]. Resolution of steroid-refractory myocarditis from nivolumab with abatacept has been demonstrated in a case report [53]. Similarly, abatacept improved myocarditis from pembrolizumab in another case report, however, evidence is limited as the patient also received plasmapheresis [54]. Abatacept being a CTLA-4 agonist induces T cell anergy leading to suppression of inflammation, however, risks include infections and possible progression of cancer [53]. Alemtuzumab has been shown to have improved steroid refractory myocarditis from PD-1 inhibitor in a case report [55]. Alemtuzumab acts by killing peripheral immune cells however does not affect the bone marrow clone of immune cells [55]. Tocilizumab has also shown efficacy in the treatment of PD-1-associated myocarditis [56, 57]. Finally, plasmapheresis helps reduce inflammation by removing immune complexes and should be considered in life-threatening cases [58].

### Outcome

In a systematic review of 99 cases, the overall case fatality rate was 35%. Mir et al reported that complete heart block and ventricular arrhythmias are associated with poor prognosis; interestingly, steroids showed no difference in survival. These patients were managed with immunosuppressive therapies, including MMF, ATG, and intravenous immunoglobulin (IVIG) to rescue 75% (9 out of 12 cases) [59]. Another systematic review reported that 31.1% of patients did not require hospitalization and the case fatality rate was 47.2%. Normalization of EKG correlated with clinical improvement [60, 61].

### Role of Re-Challenging With ICI Following ICI-Associated Myocarditis

Hasson et al, described three cases of lung adenocarcinoma in which patients were treated with durvalumab, pembrolizumab, and atezolizumab, respectively [37]. In this case series, all three patients were diagnosed with myocarditis and treated with Prednisone. Two patients could sustain the ICI re-challenge upon resolution of myocarditis. However, unlike the other patients who had grade 1 and grade 2 severity, the third patient suffered a fatal outcome attributed to grade 3 severity. Guo et al described a case of stage 4 melanoma where Ipilimumab and Nivolumab were reintroduced after the resolution of atezolizumab-associated myocarditis, however, it was complicated by immune-mediated nephritis and stopped [10]. Few case reports demonstrated successful reintroduction of pembrolizumab and nivolumab [32, 62, 63]. Re-challenging was suggested in the above cases based on the response of malignancy to immunotherapy, normalization of ejection fraction, and cardiac biomarkers. Most importantly, the grade of myocarditis determines the recurrence of myocarditis, and co-treatment with low-dose maintenance prednisone may have favorable outcomes [25].

Per ASCO guidelines, it is recommended to discontinue ICI after a Grade ≥2 cardiovascular toxicity [91]. ESMO guidelines suggest permanent discontinuation of ICIs for steroid-resistant myocarditis or grade 4 myocarditis [51]. However, clinicians are posed with the challenge of making the decision of re-introducing the ICI, especially in the setting of a good tumor response. A careful multidisciplinary discussion and individualized approach in each case is warranted to make the choice. If the decision is made to re-challenge a patient, monotherapy with an alternative agent could be considered. For example, a study has demonstrated a successful rechallenge of pembrolizumab in patients who developed irAEs from combined CTLA-4 + PD-1 inhibitors. In this study, only 18% had recurrent irAEs among which none had myocarditis [64]. Similarly, a patient with pembrolizumab-induced grade 4 myocarditis was re-challenged with nivolumab at a lower dose with no recurrence [65]. In contrast, a pharmacovigilance analysis among 180 patients reported that re-challenging with the same ICI or same class ICI is associated with a lower risk of recurrent irAEs [66]. Systemic monitoring for cardiovascular symptoms, coupled with surveillance for asymptomatic disease with serial troponins and periodic cardiac imaging, is recommended [58]. Recurrence of myocarditis seems to be lower than colitis and hepatitis during re-challenge as demonstrated in a pharmacovigilance cohort study [67]. Table 2 outlines case scenarios where ICIs were reintroduced after myocarditis.

**TABLE 2:** ICI rechallenge for ICI-associated myocarditis.

Age/Sex	Malignancy	Co-morbidities	Treatment received	Time of onset clinical presentation	Investigations	Myocarditis management	Reintroduction outcome
60 F [18]	Stage 4 Melanoma	Unknown	Atezolizumab (840 mg IV 3 weekly) and Cobimetinib after tumor progression s/p 12 cycles of Pembrolizumab	13 days Nausea, vomiting, fatigue, mucositis	TTE-normal EF. hsTnt, CK - elevated	Methylprednisolone IV then Prednisolone over 6 weeks	Resumed Rx with two cycles of ipilimumab-plus-nivolumab 3 months after resolution of cardiac toxicities
					EKG- new T wave flattening II, III, aVF.		However stopped due to immune mediated nephritis
37 [32]	Alveolar soft part sarcoma, metastatic to the lungs	Unknown	Pembrolizumab	Cycle 13 Day 2	TTE- normal EF torsades de pointes	Prednisone taper	Re-challenge with Pembrolizumab after 2 months and continued without adverse CV events
				Dyspnea, cough, vague chest discomfort	CMRI- no LGE, T2 uptake high		
61 M [37]	Lung adeno carcinoma, stage 3b	Unknown	Durvalumab 3 cycles	8 days Myalgia, leg cramps	TTE - normal LVEF (60%)	Oral Prednisone	Durvalumab was reintroduced with low dose prednisone and ACEI. No recurrence of symptoms
					CMRI- mid-wall myocardial LGE of the septal-basal segment early gadolinium enhancement		
77 M [37]	Stage IV lung adeno carcinoma	HTN, Severe AR	Pembrolizumab (200 mg)	15 days after 2nd cycle Left hand pain	TTE- LVEF- 40% (his baseline)	Oral prednisone	Pembrolizumab was reintroduced with oral prednisone and HF treatment. No recurrence Improvement in LVEF to 50%
					Troponin and CK- Elevated		
					CMRI: LVEF of 35% with patchy myocardial LGE at the septum		
63 M [37]	Early-stage lung adeno carcinoma. Grade 3	Unknown	Atezolizumab	3 days Chest pain, SOB	TTE- severe global systolic LV dysfunction, LVEF of 35%	Oral prednisone and HF therapy	-Atezolizumab was reintroduced after 12 weeks. Pt had Sob and chest pain again, no elevated Trop/no EKG changes
					CTA- right pulmonary emboli (PE), CCTA showed mild atherosclerosis with no significant occlusion		-Treatment stopped and oral prednisone continued
					hs-Tnt and CPK- normal. BNP - high		-Pt died of disease progression
54 M [68]	Melanoma	Unknown	CTLA-4 + PD-1 inhibitors	63 days	Elevation of troponin + clinical syndrome + EKG + decline in systolic function	Glucocorticoids	Rechallenged with PD-1 inhibitor without any recurrence of irAEs
71 M [68]	Non-small cell lung carcinoma	Unknown	PD-1 inhibitor	64 days	Pathology	Glucocorticoids	Rechallenged with PD-1 inhibitor without any recurrence of irAEs
							Death by tumor progression
53 F [68]	Melanoma	Unknown	CTLA-4 + PD-1 inhibitors	46 days	Elevation of troponin + clinical syndrome + concomitant myositis	Glucocorticoids, Intravenous immunoglobulin, Methotrexate, Plasma Exchange	Rechallenged with PD-1 inhibitor without any recurrence of irAEs
							Death by tumor progression
69 F [68]	Melanoma	Unknown	CTLA-4 + PD-1 inhibitors	21 days	Elevation of troponin + clinical syndrome + EKG + negative angiography	Glucocorticoids	Rechallenge with PD1 inhibitor without any recurrence of irAEs
71 M [68]	Melanoma	Unknown	CTLA-4 + PD-1 inhibitors	39 days	Elevation of troponin + clinical syndrome + EKG + elevated T2m on CMR	Glucocorticoids	Rechallenge with PD1 inhibitor without any recurrence of irAEs
72 M [68]	Non-small cell lung carcinoma	Unknown	PD-1 inhibitor	58 days	Elevation of troponin + clinical syndrome + EKG + negative angiography	None	Rechallenge with PD1 inhibitor with clinically significant relapse of myocarditis in 28 days, treated with glucocorticoids with favorable outcome
							Death by tumor progression after 6 months
85 M [65]	Gastric Adenocarcinoma	CAD with PCI, mildly reduced EF at 50%, Atrial Flutter Lynch syndrome (colorectal carcinoma, urothelial cancers, skin cancers)	Pembrolizumab	After second infusion	Troponin and pro- BNP elevated	High dose corticosteroids lead to recurrence during steroid taper	Dose reduced Nivolumab without any recurrence of irAEs
				Chest pain, SOB	EKG – sinus rhythm with PVC, non-specific ST-T abnormalities	Infliximab + concurrent methylprednisolone with resolution	
					TTE- LVEF 47% with regional wall motion abnormalities		
					CMRI- Delayed enhancement		

## Pericarditis

Pericardial disease from ICIs ranges from simple inflammation to fatal tamponade. Also, peri myocarditis/myopericarditis are other presentations associated with ICI [69].

ICI-associated pericarditis probably results from ICI-stimulated T cells against the pericardium leading to inflammation. The majority of these cases are associated with lung cancer patients [70]. The hypothesis behind this disproportionately high incidence of pericardial disease in lung cancer patients is the exposure of patients previously to radiotherapy. Theoretically, radiotherapy might expose the pericardial antigens leading to enhanced T-cell binding and inflammation [71]. Furthermore, CD 68+ve cells in pericardial fluid suggested macrophage impairment as a predisposing factor [72].

Hemodynamically significant pericardial effusions were reported in 0.38% (15/3,966) of patients treated with ICI [73]. The highest prevalence was noticed with Nivolumab (0.61%) thus far followed by pembrolizumab (0.19%) and atezolizumab (0.32%) [73]. An international database of patient case reports (WHO Vigibase) showed the average time to emergence of ICI-associated pericarditis as 30 days with a mortality of 20% [71]. A pharmacovigilance study showed that clinicians diagnosed 81% of pericarditis cases as high grade on presentation [70].

Clinical presentation of pericarditis could be cardiac symptoms like chest pain and SOB or non-specific ones such as myalgia and fatigue. EKG findings are usually non-specific, and TTE/MRI shows pericardial effusion and inflammation respectively. Also important is distinguishing pericarditis or pericardial effusion from pseudo-progression. Pseudo-progression is a condition with transient worsening of tumor status before resolution [74]. This pseudo-progression likely arises from the extensive inflammation generated from activated T cells against the tumor that leads to effusion [75, 76].

### Histopathology

In most cases, pericardiocentesis revealed malignant cells along with inflammatory cells, commonly lymphocytes [77]. However, in a retrospective study on 15 patients with ICI-associated pericardial effusion, less than a third of patients had inflammatory cells in the pericardial fluid [73].

### Grades of Pericarditis: (NCI-CTCAE v. 5.0)

G1: Asymptomatic with EKG or physical exam (example: pericardial rub) consistent with pericarditis.

G2: Symptomatic pericarditis (example: chest pain)

G3: Pericarditis with physiologic consequences.

G4: Pericarditis with life-threatening consequences needing urgent intervention G5: Death.

### Management

A widely adopted treatment strategy that has proven effective in most instances was the temporary suspension of ICI along with the use of pericardiocentesis and corticosteroids. Additionally, successful results were reported with other treatments like MMF and TNF-alpha inhibitors. Corticosteroids alone were also associated with significant improvement [39]. Intrapericardial bleomycin (bleomycin: 15 mg/kg) [60], and pericardiectomy [55] were other commonly reported procedures. A systematic review of 28 cases of pericarditis showed that around 90% of Grade 3 to Grade 4 cases needed pericardiocentesis or pericardial window in addition to corticosteroids [78]. Based on the available evidence, ICIs could be continued for Grade 1 pericarditis. Corticosteroids are the cornerstone for Grade 2 to Grade 4 pericarditis and pericardiocentesis for moderate to large effusion. However, studying the role of non-steroidal anti-inflammatory drugs (NSAIDs) and Colchicine in ICI pericarditis is imperative. Table 3 outlines case scenarios where ICIs were reintroduced after pericarditis.

**TABLE 3:** ICI rechallenge for ICI associated pericarditis.

Age/Sex	Malignancy	Prior radiotherapy/comorbidities	Immuno therapy	Type of toxicity/Time of onset	Clinical presentation	Diagnostic tests	Management	Reintroduction and outcome
65 M [79]	Lung adeno carcinoma	5 cycles of radiotherapy	Nivolumab 3 mg/kg every 2 weeks	-Pericarditis	Respiratory failure	TTE- massive pericardial effusion with tamponade	Surgical drainage and corticosteroids for 3 months	Resumed immunotherapy after 16 months without recurrence of pericarditis
				-After 35th infusion		Pericardial biopsy**-** mostly CD 4 T cell infiltrate		
70 M [80]	Stage IV lung adeno carcinoma (cT3N3M1b)	None	Pembrolizumab	-Pericardial effusion	SOB and fatigue	Chest CT- massive pericardial effusion	Pericardiocentesis	Resumed pembrolizumab after clinical improvement
						EKG- a low QRS voltage and complete RBBB		
				-After six cycles		TTE- Cardiac tamponade		No further issues
						Pericardial fluid analysis- total cell count- 4625/μL with 26% mononucleocytes and 27% polymorphonucleocytes. Cytology showed adenocarcinoma		
62 M [60]	Advanced lung adenocarcinoma	Stage IIB lung squamous cell carcinoma (cT3N0M0)	Nivolumab	-Pericardial effusion induced by pseudo-progression of immune therapy	SOB	Pericardial fluid cytology -revealed malignant cells	-Emergency pericardiocentesis	Restarted nivolumab without other irAE
				-After 7days			-cardiac tamponade with dyspnea 6 weeks after pericardiocentesis	
							-intrapericardial bleomycin	
70 F [62]	Metastatic adenocarcinoma of lung	Chemoradiation	Nivolumab	-Pericardial effusion	SOB and chest pain	EKG- showed sinus tachycardia	-Prednisone	Nivolumab resumed
				-Four days after first dose		TTE- large pericardial effusion	Continued to receive nivolumab but discontinued due to worsening effusion	No recurrence of effusion
						Pericardial fluid cytology-showed 5% lymphocytes		
46 M [75]	Small cell lung cancer	Concurrent radiation therapy h/o pleural effusions- present	Nivolumab (3 mg/kg every 2 weeks)	-Pericardial effusion with tamponade	Not known	Pericardial fluid cytology- showed 5% lymphocytes	Pericardiocentesis	Treatment was continued every 2 weeks without any break. No further progression of effusion
				-Week 9				
57 M [72]	Metastatic lung adenocarcinoma	No prior RT	Anti–PD-L1 therapy	-Cardiac tamponade	SOB, orthopnea, bilateral lower limb edema	Unknown	Pericardial window procedure	Re-challenged with the same immunotherapy
				−98 days after the first dose				No dose-limiting toxicities
70 F [39]	Metastatic lung adenocarcinoma	Chemoradiation	nivolumab	-Pericardial effusion	SOB, chest pain	EKG- sinus tachycardia	Prednisone	Nivolumab resumed
				−4 days after first dose		TTE- large pericardial effusion		No recurrence of effusion
						Cytology- 5% lymphocytes		
65 M [81]	Adenocarcinoma of the lung stage IVB	No prior RT	Nivolumab (3 mg/kg every 2 weeks)	-Cardiac tamponade	SOB	TTE- massive pericardial effusion	Pericardiocentesis	Nivolumab therapy was continued
				-After four cycles		Fluid analysis and cytology- Lymphocyte predominant and adenocarcinoma		No recurrence
63 F [82]	Stage IVA lung adeno carcinoma	No prior RT	pembrolizumab (200 mg/kg bodyweight, triweekly)	-Cardiac tamponade	SOB	CT chest- massive pericardial and bilateral pleural effusions	Pericardiocentesis	Resumed Pembrolizumab
				-After four cycles		TTE- cardiac tamponade		Stable clinical condition
						Cytology- no malignant cells, predominant neutrophils and few lymphocytes		

## Atherosclerosis & Dyslipidemia

Multiple animal and human studies have demonstrated that PD-1, PD-L1, and CTLA-4 are down regulators of atherosclerosis [83, 84]. PD-1 deficiency could potentially increase cholesterol synthesis via gene regulation [85]. A meta-analysis has shown a significant association between ICIs and dyslipidemia leading to atherosclerosis and myocardial infarction (MI). Interestingly, dyslipidemia is this study’s most common adverse effect [86]. A single-center retrospective study has shown a three-fold higher risk of aortic plaque progression and coronary atherosclerosis leading to myocardial infarction, ischemic stroke, and coronary revascularization [4]. Another study demonstrated enhanced FDG-PET uptake in large arteries after ICI treatment signifying a pro-inflammatory state [87]. The above studies did not comment on previous radiotherapy administration, making the results questionable. Due to the probable association between ICIs and inflammation leading to atherosclerosis, treatment options to reduce this risk are of priority. Drobni et al revealed that the progression of aortic plaque while on ICIs was diminished with the usage of statin [4]. Statins enhance antigen presentation to CD4^+^ and CD8^+^ T cells by reducing protein prenylation in mice models [88]. This confers an enhanced inflammatory response and might lead to a synergistic action with ICIs. Notably, omori et al and cantini et al demonstrated increased response rate, PFS, and OS with statins while on anti-PD-1 therapy [89, 90]. A single-center retrospective observational study by Buti et al evaluated the effect of concomitant medications with ICI initiation on overall response rate (ORR), progression-free survival (PFS), and overall survival (OS). Statins were associated with better OS [91]. Another observational retrospective study by cortellini et al analyzed oncologic outcomes with ICIs while on concomitant medications, statins were associated with a better objective response rate [92]. A meta-analysis of cantini et al, buti et al, cortellini et al, and two other studies where statins and ICIs were used concomitantly, revealed better overall PFS and OS. Sub-group analysis, however, showed a correlation for better OS and PFS in cases where PD-1/PD-L1 inhibitors are used [93–95]. Similar to statins, PCSK-9 inhibitors also demonstrated synergistic action with PD-1 inhibitors by suppressing tumor growth in mouse models [96]. Further studies are necessary to validate the synergistic effect of statins and PCSK-9 inhibitors with ICIs. In addition, establishing the safety profile of statins while on ICIs is crucial due to the risk of adverse effects including but not limited to myopathy.

## Arrhythmias

ICIs can lead to arrhythmias such as atrial fibrillation, supraventricular tachycardias, ventricular tachycardia, and heart blocks. It is important to note that these arrhythmias may arise independently due to various factors, such as concurrent myocarditis, pericarditis, or electrolyte abnormalities, and their relationship to ICIs is non-overlapping [70]. Ventricular arrhythmias are commonly seen in underlying myocarditis, making a correlation between ICIs and ventricular arrhythmias challenging [97].

## Heart Failure & Cardiomyopathy

Cardiomyopathy resulting from ICIs can take two forms: inflammatory cardiomyopathy, typically associated with underlying myocarditis and non-inflammatory cardiomyopathy. Researchers hypothesize that the non-inflammatory heart failure syndrome is a delayed side effect [1]. Non-inflammatory cardiomyopathy presents as an exclusionary diagnosis where troponin is normal and there is no inflammation on CMRI [98]. Other presentations include Takotsubo cardiomyopathy [99]. The use of corticosteroids to treat non-inflammatory heart failure syndrome appears moot due to lack of inflammation, but further studies are necessary.

## Vasculitis

Researchers hypothesize that the etiology of vasculitis stemming from ICIs comes from augmented inflammation within the blood vessels. For instance, in a study focused on Giant Cell Arteritis (GCA), it was observed that the expression levels of PD-1 and PD-L1 were diminished in the affected temporal arteries [100]. This indicates a potential link between ICIs and the onset of vasculitis. The deficiency of PD-1 and PD-L1 contributes to excess cytokine and T-cell aggregation response [101]. A pharmacovigilance study attributed 0.2% of irAEs to vasculitis, among other cardiotoxicities. Temporal arteritis was the most commonly reported event [70]. A case series reported that ICI-related vasculitis mainly involves large arteries, especially in the central nervous system [102]. Treatment mainly involves corticosteroids [103].

## Arterial and Venous Thrombosis

Researchers hypothesize that a deficiency of PD-1, PD-L1, and CTLA-4 leads to enhanced inflammation culminating in arterial thrombosis (ATE) and venous thrombosis (VTE) [104]. A systematic review reported a VTE rate of 2.7% and an ATE rate of 1.1% [105]. In contrast, a recent meta-analysis did not report an increased risk of VTE in patients treated with ICIs [106]. Caution has to be exercised in attributing ICIs as the cause of thrombosis, as underlying cancer or paraneoplastic syndrome could be the primary driver of thrombosis. At this time, the role of prophylactic low-dose anticoagulation is unclear in patients treated with it. Further studies are warranted to develop a scoring system exclusively for ICI-treated patients to predict the necessity of prophylactic anticoagulation. ASCO guidelines stratify ICI-associated VTE into four grades [50].

G1: Superficial venous thrombosis.

G2: Uncomplicated deep vein thrombosis (DVT).

G3: Uncomplicated pulmonary embolism (PE).

G4: Life-threatening consequences from DVT or PE.

For G1, ASCO recommends continuing ICIs and close clinical surveillance. They recommend discontinuing ICIs and starting anticoagulation for G2 to G4 VTE. Re-initiation of ICIs might be an option after considering risks and benefits in the future for G2 to G4 toxicities [50].

## Conclusion

Recognizing patients at risk, their ongoing monitoring and the chance to reintroduce immunotherapy presents significant challenges, particularly for those with a terminal prognosis. The evidence supporting the re-administration of ICIs for patients who experienced cardiac toxicity is scarce, and there is a lack of expert guidance in this area. Early and continuous interdisciplinary collaboration with cardiologists and oncologists is crucial to manage this situation effectively. Further research is required to understand the roles of cardiac MRIs and FDG-PETs, particularly given the limitations associated with invasive biopsy techniques. Besides myocarditis and pericarditis, it is important to identify and manage other potential ICI-related complications such as atherosclerosis, heart failure, and myocardial infarction. This attention to detail is vital in creating a comprehensive strategy for ICI-related cardiac events.

## Supplementary Material

Supplementary Material S1 (A) Main messages, (B) Multiple choice questions, (C) Key references, (D) Current research questions, (E) Answers.
