# Supplementary material for: Immune Checkpoint Inhibitors and Their Cardiovascular Adverse Effects
Source: Oncol Rev. 2023 Nov 17;17:11456. doi: 10.3389/or.2023.11456 (PMC10691592; doi:10.3389/or.2023.11456)
Supplement: Supplementary file 1 [file DataSheet1.docx]

**Main Messages:**

- ICI-related cardiotoxicity has varied clinical manifestations with myocarditis, pericarditis, cardiomyopathy and arrhythmias being the commonly described ones.
- EKG, Troponin, TTE, and cardiac MRI are indispensable elements in the diagnosis of ICI-related cardiotoxicity.
- Evidence to re-challenge ICI after cardiotoxicity is limited. Re-challenging is done based on the response of malignancy to immunotherapy, normalization of EF, cardiac biomarkers, and most importantly the grade of myocarditis.

**Multiple Choice Questions (true(T)/false(F)):**

Question 1:

1. CTLA-4, and PD-1 are activating co-stimulatory molecules.
2. Ipilimumab is a monoclonal antibody acting against CTLA-4.
3. CTLA-4 and PD-1 affect the same stage of immune tolerance.
4. PD-1 binds with PDL1 on cardiac myocytes.

Question 2:

1. EKG and troponin are helpful in the diagnosis of ICI-associated myocarditis
2. Myocarditis and pericarditis are the only manifestations of ICI-associated cardiotoxicity
3. Late gadolinium enhancement on cardiac MRI is a feature of ICI-associated myocarditis
4. There are typically four grades of myocarditis.

Question 3:

1. Steroids are a cornerstone in the management of ICI-associated myocarditis
2. Steroids improve prognosis in all cases of ICI-associated myocarditis
3. Immunosuppressive agents such as MMF/Tacrolimus have been used in some cases of myocarditis due to ICIs
4. Ventricular arrhythmias/high grade AV block are poor prognostic signs in ICI associated myocarditis.

Question 4:

1. Pericarditis is a well-recognized complication of ICI-associated cardiotoxicity
2. Chest pain and shortness of breath are common presenting features of ICI-associated pericarditis
3. Pericardial effusion from pseudo-progression of tumor is different from pericardial effusion as a complication of ICI associated pericarditis.
4. Cardiac tamponade is a known complication of ICI-associated pericarditis.

Question 5:

1. EKG and troponin are commonly used to monitor for recurrence of myocarditis after re-challenging ICI’s
2. Corticosteroids are commonly employed in treating ICI-associated pericarditis
3. T cells are the major cells found on endomyocardial biopsy of patients with ICI-associated myocarditis
4. Autoimmune diseases might increase the risk of ICI-associated cardiotoxicity.

**Key References:**

Guo, C.W., et al., *A closer look at immune-mediated myocarditis in the era of combined checkpoint blockade and targeted therapies.* Eur J Cancer, 2020. **124**: p. 15-24.

Schneider, B.J., et al., *Management of Immune-Related Adverse Events in Patients Treated With Immune Checkpoint Inhibitor Therapy: ASCO Guideline Update.* J Clin Oncol, 2021. **39**(36): p. 4073-4126.

Peleg Hasson, S., et al., *Reintroducing immunotherapy in patients surviving immune checkpoint inhibitors-mediated myocarditis.* Clin Res Cardiol, 2020.

Saade, A., et al., *Pericardial effusion under nivolumab: case-reports and review of the literature.* J Immunother Cancer, 2019. **7**(1): p. 266.

Pradhan, R., A. Nautiyal, and S. Singh, *Diagnosis of immune checkpoint inhibitor-associated myocarditis: A systematic review.* Int J Cardiol, 2019. **296**: p. 113-121.

**Current research questions:**

- Does type of cancer being treated predict the risk of development of ICI associated cardiotoxicity
- Developing a standardized model for predicting the prognosis of ICI associated myocarditis.
- Safety of re-challenging immune checkpoint inhibitors in myocarditis grade 3 and above.

**Answers:**

A(F); B(T); C(F); D(T)

A(T); B(F); C(T); D(T)

A(T); B(F); C(T); D(T)

A(T); B(T); C(T); D(T)

A(T); B(T); C(T); D(T).
